# Supplementary material for: Understanding Complex Interplay among Different Instabilities in Multiferroic BiMn7O12 Using 57Fe Probe Mössbauer Spectroscopy
Source: Int J Mol Sci. 2024 Jan 24;25(3):1437. doi: 10.3390/ijms25031437 (PMC10855744; doi:10.3390/ijms25031437)
Supplement: Supplementary file 1 [file ijms-25-01437-s001.zip › ijms-2834461-supplementary.pdf]

Supplementary Materials

Understanding Complex Interplay among Different Instabilities in Multiferroic $BiMn_7O_{12}$ Using ^{57}Fe Probe Mössbauer Spectroscopy

Iana S. Soboleva [1], Vladimir I. Nitsenko [1], Alexey V. Sobolev [1,2,*], Maria N. Smirnova [1,3], Alexei A. Belik [4] and Igor A. Presniakov [1,2]

[1] Department of Chemistry, Lomonosov Moscow State University, Moscow 119991, Russia
[2] Department of Chemistry, MSU-BIT University, Shenzhen 517182, China
[3] Kurnakov Institute of General and Inorganic Chemistry of Russian Academy of Sciences (RAS), Moscow 119991, Russia
[4] Research Center for Materials Nanoarchitectonics (MANA), National Institute for Materials Science (NIMS), Namiki 1-1, Tsukuba 305-0044, Ibaraki, Japan
* Correspondence: alex@radio.chem.msu.ru

Table S1. Calculated Born charges *Z* and average effective charges of ions <*S*> in the structural modifications of $BiMn_7O_{12}$.

Structural Modification	Atom	*Z*	<*S*>
Im ($T_3 < T < T_2$)	Bi	+3.30	+2.55
	Mn	+3.30	+3.03
	O	-2.20	-1.99
*P*1 ($T < T_3$)	Bi	+4.50	+2.45
	Mn	+4.50	+3.08
	O	-3.30	-2.00

Table S2. ^{57}Fe hyperfine parameters at $T > T_1$ of $BiMn_{6.96}{}^{57}Fe_{0.04}O_{12}$. <$\delta$> - is the average isomer shift, <Δ> - is the average quadruple splitting, D_p^{exp} – is the dispersion of the experimental distribution $p(\Delta)$, Γ – is linewidth.

***T*, K**	**<δ>, mm/s**	**<Δ>, mm/s**	**D_p^{exp}, mm²/s²**	**Γ, mm/s**
602	0.18(1)	0.28(1)	0.016(1)	0.24*
612	0.18(1)	0.27(1)	0.016(1)	0.24*
622	0.17(1)	0.26(1)	0.016(1)	0.24*
633	0.16(1)	0.26(1)	0.017(1)	0.24*
643	0.16(1)	0.26(1)	0.017(1)	0.24*
653	0.15(1)	0.25(1)	0.016(1)	0.24*
669	0.13(1)	0.25(1)	0.017(1)	0.24*

* When processing the spectra, the linewidth Γ was fixed.

Table S3.1. ^{57}Fe hyperfine parameters at $T_2 < T < T_1$ of $BiMn_{6.96}{}^{57}Fe_{0.04}O_{12}$. δ – is the isomer shift, $\ln(n_1/n_2)$ – is logarithm of probabilities n_1 and n_2 ratio, $\ln(\Omega_R)$ - is logarithm of the average relaxation frequency.

***T*, K**	**δ, mm/s**	**$\ln(n_1/n_2)$**	**$\ln(\Omega_R)$**
448	0.285(1)	1.83(33)	16.43(1)
458	0.278(1)	1.76(42)	16.58(1)
468	0.272(1)	1.73(49)	16.64(1)
479	0.265(1)	1.69(39)	16.76(1)
489	0.256(1)	1.69(37)	16.88(1)
499	0.249(1)	1.62(35)	16.91(1)
510	0.244(1)	1.60(35)	17.03(1)
520	0.237(1)	1.52(35)	17.12(1)
530	0.227(1)	1.49(29)	17.19(1)
540	0.224(1)	1.40(35)	17.37(1)
550	0.215(1)	1.36(31)	17.42(1)
561	0.209(1)	1.27(31)	17.56(1)
571	0.203(1)	1.21(32)	17.66(1)
581	0.196(1)	1.08(25)	17.79(1)
592	0.189(1)	0.95(31)	17.91(1)

Table S3.2. ^{57}Fe hyperfine parameters at $T_2 < T < T_1$ of $BiMn_{6.96}{}^{57}Fe_{0.04}O_{12}$ obtained in static doublet model. δ – is the isomer shift value, Δ - is the quadruple splitting value, Γ – is linewidth.

***T*, K**	***δ*, mm/s**	**Δ, mm/s**	***Γ*, mm/s**
448	0.285(1)	0.531(2)	0.31(1)
458	0.278(1)	0.517(3)	0.31(1)
468	0.272(1)	0.509(3)	0.31(1)
479	0.265(1)	0.502(1)	0.30(1)
489	0.256(1)	0.499(2)	0.29(1)
499	0.249(1)	0.487(2)	0.30(1)
510	0.244(1)	0.479(2)	0.29(1)
520	0.237(1)	0.465(1)	0.30(1)
530	0.227(1)	0.457(2)	0.29(1)
540	0.224(1)	0.434(2)	0.29(1)
550	0.215(1)	0.425(2)	0.29(1)
561	0.209(1)	0.401(1)	0.29(1)
571	0.203(1)	0.386(2)	0.28(1)
581	0.196(1)	0.352(1)	0.28(1)
592	0.189(1)	0.315(2)	0.29(1)

Table S4. ^{57}Fe hyperfine parameters at $T_{N1} < T < T_2$ of BiMn$_{6.96}{}^{57}$Fe$_{0.04}$O$_{12}$. δ – is the isomer shift value, Δ - is the quadruple splitting value, Γ – is linewidth.

***T*, K**	**Position**	**δ, mm/s**	**Δ, mm/s**	**Γ, mm/s**
101	Mn5	0.489(1)	1.13(1)	0.27(1)
	Mn4+Mn6		0.86(1)	
	Mn7		0.55(1)	
150	Mn5	0.468(2)	1.09(1)	0.27(1)
	Mn4+Mn6		0.83(1)	
	Mn7		0.52(1)	
180	Mn5	0.452(1)	1.04(1)	0.29(1)
	Mn4+Mn6		0.81(1)	
	Mn7		0.52(1)	
190	Mn5	0.447(2)	1.02(1)	0.29(1)
	Mn4+Mn6		0.80(1)	
	Mn7		0.53(1)	
200	Mn5	0.441(1)	1.00(1)	0.29(1)
	Mn4+Mn6		0.79(1)	
	Mn7		0.52(1)	
209	Mn5	0.435(1)	0.99(1)	0.30(1)
	Mn4+Mn6		0.78(1)	
	Mn7		0.52(1)	
240	Mn5	0.408(2)	0.95(1)	0.29(1)
	Mn4+Mn6		0.75(1)	
	Mn7		0.51(1)	
300	Mn2	0.376(1)	0.77(1)	0.31(1)
	Mn1		0.55(1)	
355	Mn2	0.344(2)	0.72(1)	0.32(1)
	Mn1		0.51(1)	
396	Mn2	0.317(1)	0.68(1)	0.30(1)
	Mn1		0.47(1)	
427	Mn2	0.296(1)	0.64(1)	0.29(1)
	Mn1		0.46(1)	
437	Mn2	0.287(6)	0.59(3)	0.32(1)
	Mn1		0.49(4)	

448	Mn4+Mn5	0.285(1)	0.531(2)	0.31(1)
458	Mn4+Mn5	0.278(1)	0.517(3)	0.31(1)
468	Mn4+Mn5	0.272(1)	0.509(3)	0.31(1)
479	Mn4+Mn5	0.265(1)	0.502(1)	0.30(1)
489	Mn4+Mn5	0.256(1)	0.499(2)	0.29(1)
499	Mn4+Mn5	0.249(1)	0.487(2)	0.30(1)
510	Mn4+Mn5	0.244(1)	0.479(2)	0.29(1)
520	Mn4+Mn5	0.237(1)	0.465(1)	0.30(1)
530	Mn4+Mn5	0.227(1)	0.457(2)	0.29(1)
540	Mn4+Mn5	0.224(1)	0.434(2)	0.29(1)
550	Mn4+Mn5	0.215(1)	0.425(2)	0.29(1)
561	Mn4+Mn5	0.209(1)	0.401(1)	0.29(1)
571	Mn4+Mn5	0.203(1)	0.386(2)	0.28(1)
581	Mn4+Mn5	0.196(1)	0.352(1)	0.28(1)
592	Mn4+Mn5	0.189(1)	0.315(2)	0.29(1)

Table S5. ^{57}Fe hyperfine parameters at $T_{N1} < T < T_1$ of $BiMn_{6.96}{}^{57}Fe_{0.04}O_{12}$. δ – is the isomer shift value, Δ - is the quadruple splitting value, Γ – is linewidth.

***T*, K**	**Position**	***δ*, mm/s**	**Δ, mm/s**	***Γ*, mm/s**
101	Mn5	0.489(1)	1.13(1)	0.27(1)
	Mn4+Mn6		0.86(1)	
	Mn7		0.55(1)	
150	Mn5	0.468(2)	1.09(1)	0.27(1)
	Mn4+Mn6		0.83(1)	
	Mn7		0.52(1)	
180	Mn5	0.452(1)	1.04(1)	0.29(1)
	Mn4+Mn6		0.81(1)	
	Mn7		0.52(1)	
190	Mn5	0.447(2)	1.02(1)	0.29(1)
	Mn4+Mn6		0.80(1)	
	Mn7		0.53(1)	
200	Mn5	0.441(1)	1.00(1)	0.29(1)
	Mn4+Mn6		0.79(1)	
	Mn7		0.52(1)	
209	Mn5	0.435(1)	0.99(1)	0.30(1)
	Mn4+Mn6		0.78(1)	
	Mn7		0.52(1)	
240	Mn5	0.408(2)	0.95(1)	0.29(1)
	Mn4+Mn6		0.75(1)	
	Mn7		0.51(1)	
300	Mn2	0.376(1)	0.77(1)	0.31(1)
	Mn1		0.55(1)	
355	Mn2	0.344(2)	0.72(1)	0.32(1)
	Mn1		0.51(1)	
396	Mn2	0.317(1)	0.68(1)	0.30(1)
	Mn1		0.47(1)	
427	Mn2	0.296(1)	0.64(1)	0.29(1)
	Mn1		0.46(1)	
437	Mn2	0.287(6)	0.59(3)	0.32(1)
	Mn1		0.49(4)	

Table S6. ^{57}Fe hyperfine parameters at $T < T_N$ of $BiMn_{6.96}{}^{57}Fe_{0.04}O_{12}$. $<\varepsilon>$ - is the average quadruple shift value, D_p^{exp} – is the dispersion of the experimental distribution $p(\delta)$, $<B_{hf}>$ – is the average value of hyperfine magnetic field.

***T*, K**	**$<\varepsilon>$, mm/s**	**D_p^{exp}, mm^2/s^2**	**$<B_{hf}>$, T**
11	$3.2(9)\cdot 10^{-2}$		46.3(2)
32	$4(1)\cdot 10^{-2}$		37.3(4)
50	$4(1)\cdot 10^{-2}$	10.9(1)	22.4(2)
51.4	$4(1)\cdot 10^{-2}$	8.9(2)	20.1(2)
54	$19(7)\cdot 10^{-2}$	5.6(2)	16.2(2)
57		3.0(5)	0.74(3)
60		1.5(6)	0
70		1.6(9)	0

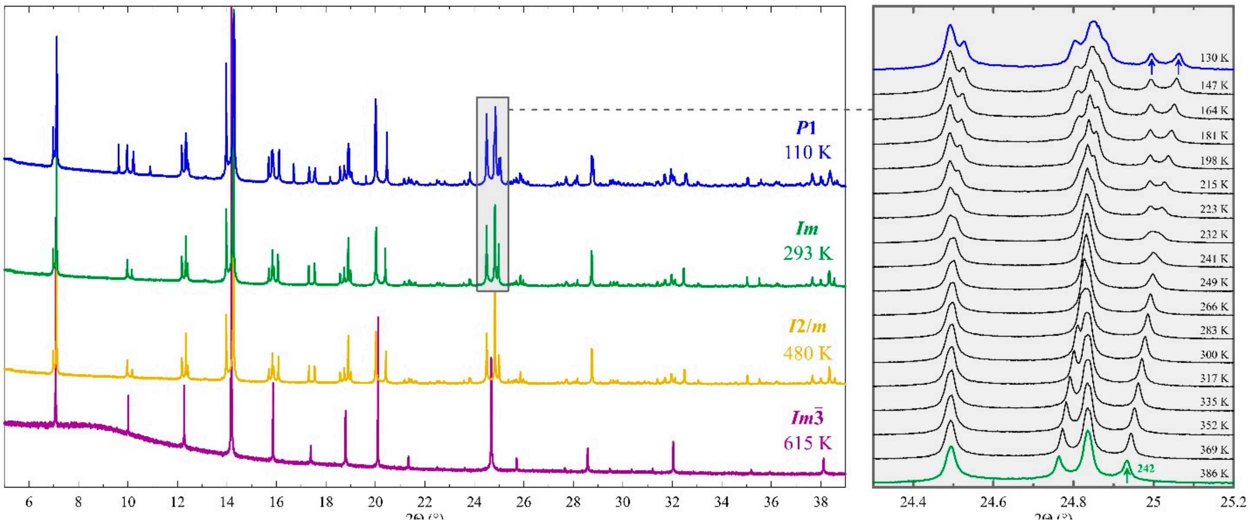

Figure S1. X-ray patterns of $BiMn_{6.96}{}^{57}Fe_{0.04}O_{12}$ manganite, measured in different temperature ranges. The right panel shows the splitting of the (242) monoclinic peak below ~ 240 K denoting the *Im* → *P*1 structural phase transition.

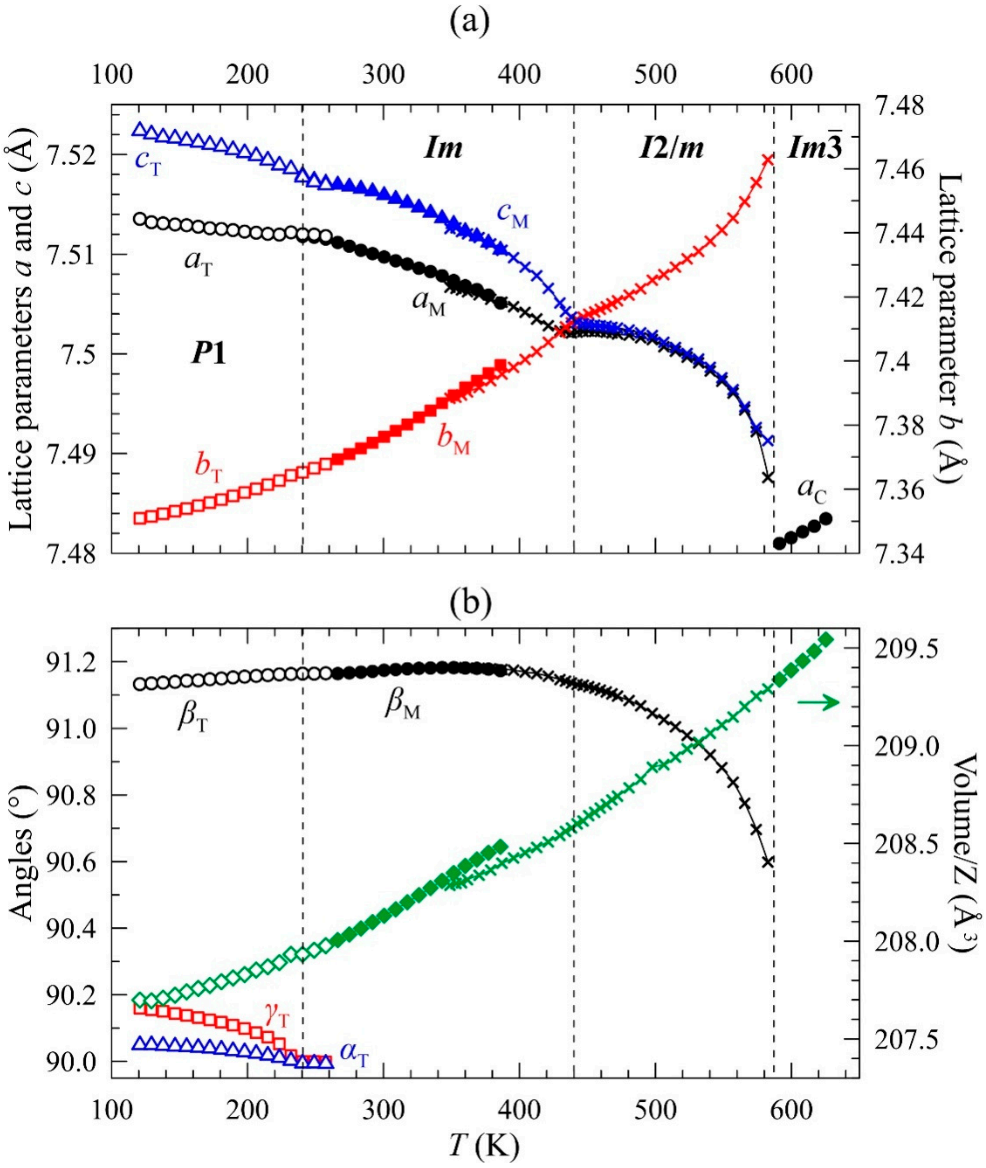

Figure S2. Temperature dependences of (**a**) crystal lattice parameters (*a*, *b*, *c*) and (**b**) angles (*α*, *β*, *γ*), and volume of the unit cells of different structural modifications of the $BiMn_{6.96}{}^{57}Fe_{0.04}O_{12}$ manganite.

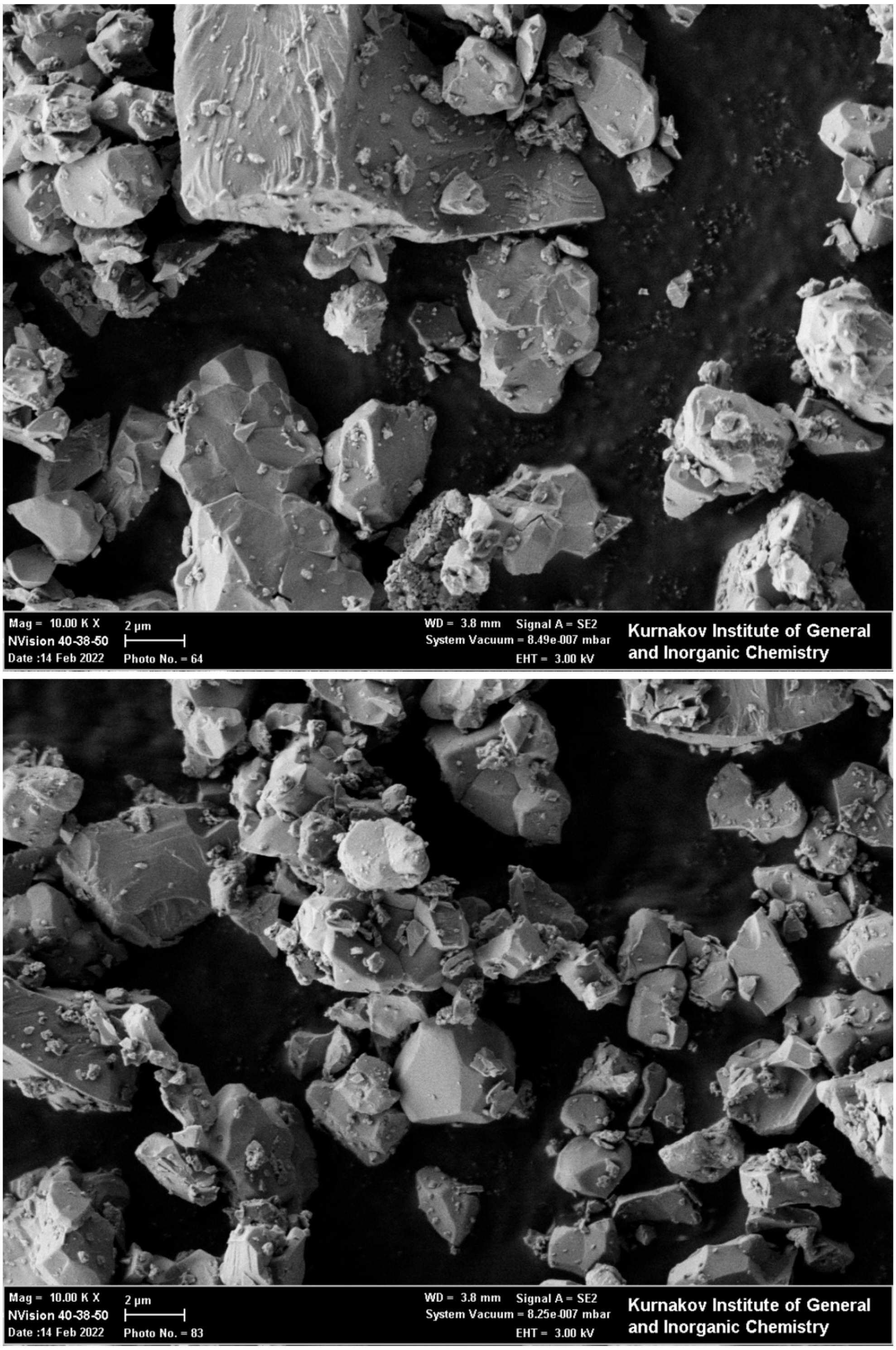

Figure S3. SEM images of the powders $BiMn_{6.96}{}^{57}Fe_{0.04}O_{12}$ in different projections.

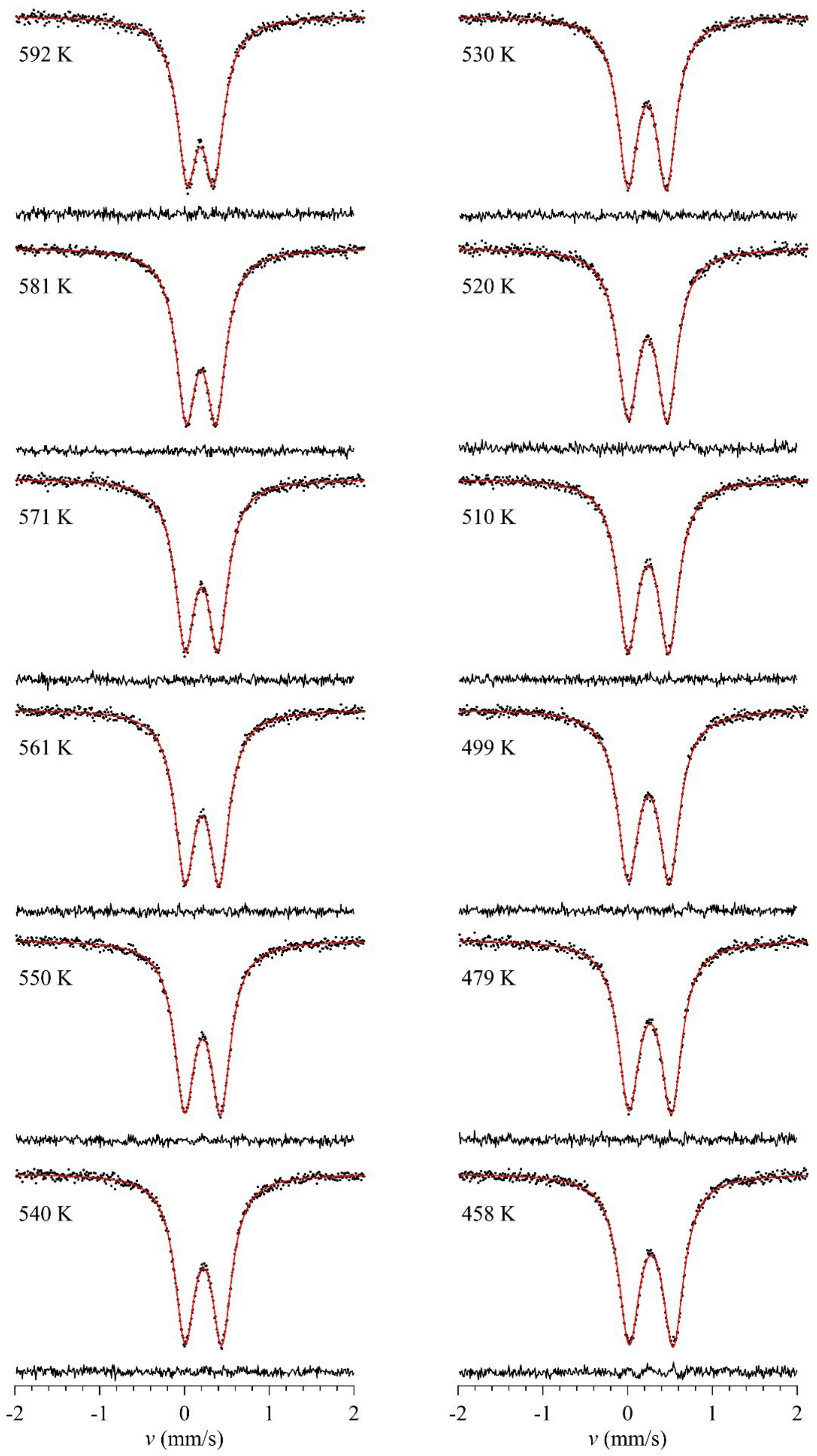

Figure S4. ^{57}Fe Mössbauer spectra of $BiMn_{6.96}{}^{57}Fe_{0.04}O_{12}$ manganite ($T_2 < T < T_1$), fitted with the TLM (two-level paramagnetic relaxation model) approach.

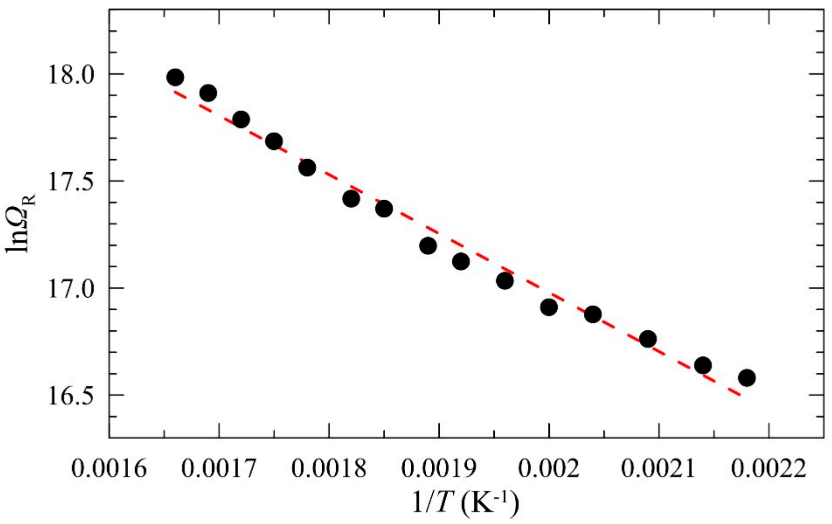

Figure S5. Reciprocal temperature dependence of the logarithm of the average relaxation frequency $\ln\Omega_R$, obtained by spectra approximation in terms of the TLM ($T_2 < T < T_1$) (see text).

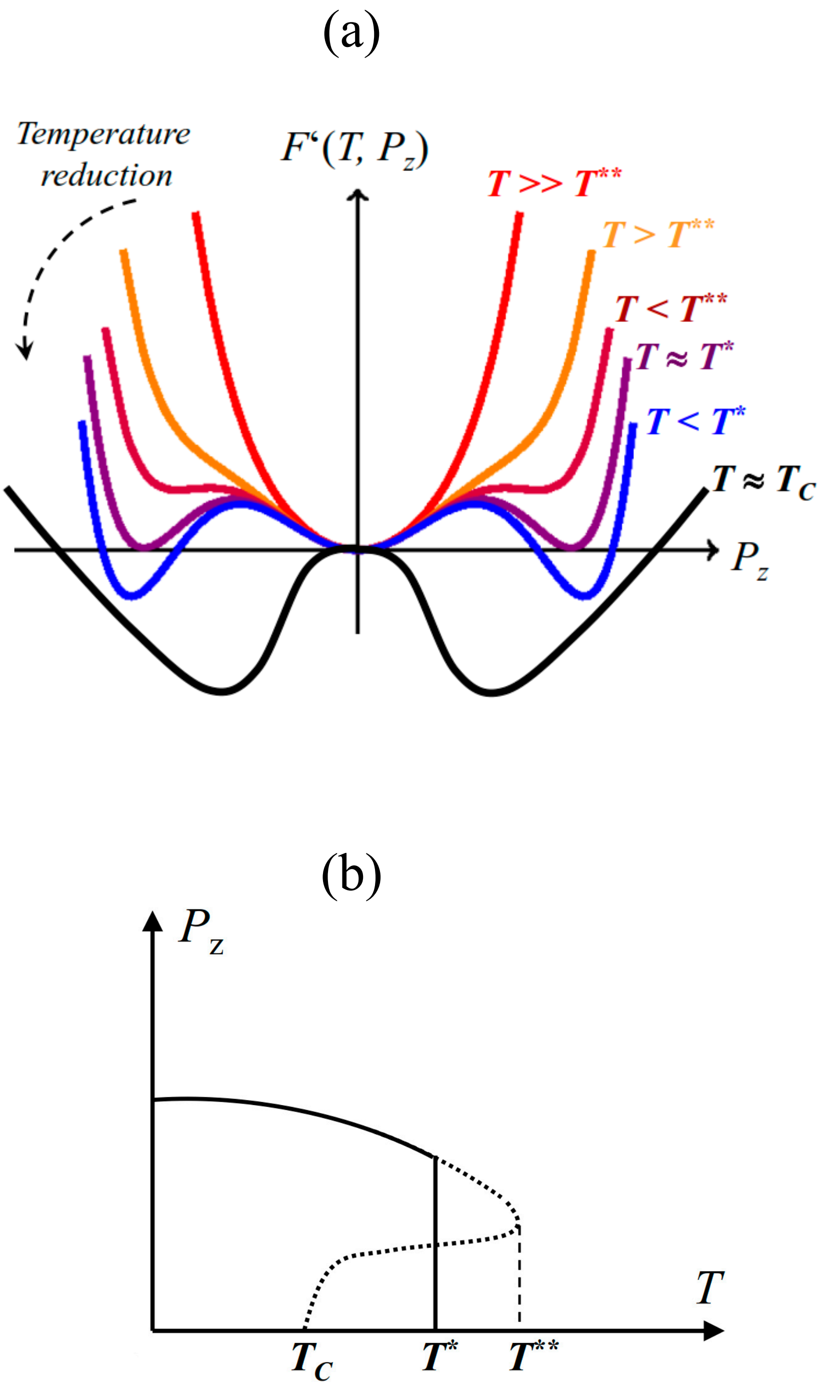

Figure S6. Temperature dependences of (**a**) the non-equilibrium Landau energy given by Equation (A9) and (**b**) the order-parameter (P_z) across a first-order transition (see text).
